# Passive Detection and Imaging of Human Body Radiation Using an Uncooled Field-Effect Transistor-Based THz Detector

**DOI:** 10.3390/s20154087

**Published:** 2020-07-22

**Authors:** Dovilė Čibiraitė-Lukenskienė, Kęstutis Ikamas, Tautvydas Lisauskas, Viktor Krozer, Hartmut G. Roskos, Alvydas Lisauskas

**Affiliations:** 1 Physikalisches Institut, J. W. Goethe University Frankfurt, 60438 Frankfurt, Germany; krozer@physik.uni-frankfurt.de (V.K.); Roskos@physik.uni-frankfurt.de (H.G.R.); 2 Institute of Applied Electrodynamics and Telecommunications, Vilnius University, 10257 Vilnius, Lithuania; kestutis.ikamas@ff.vu.lt; 3 The General Jonas Žemaitis Military Academy of Lithuania, 10322 Vilnius, Lithuania; 4 MB “Terahertz Technologies”, 01116 Vilnius, Lithuania; tautvydas.lisauskas@gmail.com; 5 Ferdinand-Braun-Institut, Leibniz-Institut für Höchstfrequenztechnik (FBH), 12489 Berlin, Germany; 6 CENTERA Laboratories, Institute of High Pressure Physics PAS, 01-142 Warsaw, Poland

**Keywords:** passive imaging, human-body radiation, THz detection, TeraFET, field-effect transistor, terahertz

## Abstract

This work presents, to our knowledge, the first completely passive imaging with human-body-emitted radiation in the lower THz frequency range using a broadband uncooled detector. The sensor consists of a Si CMOS field-effect transistor with an integrated log-spiral THz antenna. This THz sensor was measured to exhibit a rather flat responsivity over the 0.1–1.5-THz frequency range, with values of the optical responsivity and noise-equivalent power of around 40 mA/W and 42 pW/$\sqrt{\mathrm{Hz}}$, respectively. These values are in good agreement with simulations which suggest an even broader flat responsivity range exceeding 2.0 THz. The successful imaging demonstrates the impressive thermal sensitivity which can be achieved with such a sensor. Recording of a 2.3 × 7.5-cm${}^{2}$-sized image of the fingers of a hand with a pixel size of 1 mm${}^{2}$ at a scanning speed of 1 mm/s leads to a signal-to-noise ratio of 2 and a noise-equivalent temperature difference of 4.4 K. This approach shows a new sensing approach with field-effect transistors as THz detectors which are usually used for active THz detection.

## 1. Introduction

The recording of images of the human body in a passive manner, that is, using its own thermal radiation, is a well-established imaging modality in the infrared (IR) at wavelengths around 10 μm, where the black-body radiation of living beings is at its highest spectral intensity. At IR wavelengths, detectors can be operated at room temperature. Towards longer wavelengths, into the THz and sub-THz regime, the power of the Planck radiation drops strongly, with the consequence that unaided passive detection at room temperature, typically using bolometers and microbolometer-based cameras as sensors [1,2,3], is impractical. As an alternative, active illumination [4,5,6,7,8,9] has been explored in the literature.

Passive detection can be maintained at sub-THz frequencies when using superconducting sensor devices. Passive imaging with human-body radiation and penetration through various materials was shown for example at 350 GHz and 850 GHz using a detector array of 20 superconducting bolometers [10]. Even video-rate human-body imaging at 350 GHz was demonstrated with superconducting detectors cooled below 1 K [11,12,13]. While feasible for stationary security applications, cryogenic passive imaging systems do not lend themselves for most of the potential mobile applications which have been identified—the sensing of the surroundings of moving cars [14] and airplanes, the visibility through fog, clouds and smoke, and the identification of boats and oil spills on the sea, to name a few [15].

Towards millimeter-wave (mm-wave) frequencies, passive imaging with detectors based on semiconductor diodes at room temperature again becomes possible despite the even lower spectral intensity of the Planck radiation. The systems—sub-THz and mm-wave radiometers—use low-noise electronic amplifiers to boost the weak signals and often also use powerful local oscillators (LO) for frequency down-conversion to intermediate frequencies where amplification is easier [16,17,18,19,20,21,22,23,24,25,26,27,28,29]. The noise-equivalent power (NEP) of radiometer receivers in the W-band (75–100 GHz) is below 10 fW/$\sqrt{\mathrm{Hz}}$ [26,30] and can reach 0.28 fW/$\sqrt{\mathrm{Hz}}$ for special pixel designs [28]. Passive imaging of a human hand was demonstrated at 220 GHz employing a mm-wave radiometer with a Schottky diode held at room temperature [31] exhibiting a 1500-K equivalent mixer noise temperature. Going to much higher frequencies poses a challenge, as the performance of the amplifiers and mixers deteriorates. The performance degradation, expressed by the increase of the equivalent noise temperature, does not allow to work at higher THz frequencies, which is often desirable for a better spatial resolution. Sensitivity can be recovered through deep cooling of the receivers at the expense of the complexities associated with cryogenic cooling. For these reasons, it is worth to explore other detector technologies for their potential of unaided passive detection of thermal radiation. This paper focuses on TeraFETs, THz detectors based on field-effect transistors (FETs) with integrated antennas. The sensing principle is full power detection of THz emitter power. TeraFETs as sensor devices offer the advantage of being directly scalable to large arrays and hence have the capabilities of THz camera sensing systems.

Over the last years, the sensitivity of TeraFETs has continued to approach the level needed for the detection of the black-body radiation from warm and moderately hot objects while maintaining the detector itself at room temperature. Reported in 2016, the emission of a black-body cavity radiator at a temperature of 1200 K was used for video-rate imaging with a 1 k-pixel complementary metal-oxide semiconductor (CMOS) TeraFET camera [32]. Temporal integration of the image frames for 5.7 min led to a noise-equivalent temperature difference (NETD) of 21 K. With an AlGaN/GaN TeraFET cooled with liquid nitrogen to 77 K, shadow images of a toy car and a surgical knife were recorded using the thermal emission of a radiator at 773 K [33]. Remarkably, a pixel integration time of 200 ms was sufficient, which impressively documents the high sensitivity reached with the present TeraFET technology.

As a step further for this technology, we demonstrate here the first completely unaided passive imaging of a human hand with an uncooled TeraFET detector. The challenge is the very small temperature difference of less than 9 K between the palm of a hand and the room-temperature background. To our knowledge, this is the first demonstration of passive imaging with human-body radiation using quasi-optical TeraFET detectors. The enabling aspect of our approach is to work with an ultra-wide 3-dB bandwidth of more than 1.4 THz. The detector covers the frequency range of 0.1–1.5 THz, thus bridging the mm-wave regime and the lower THz range, which formerly was accessible only to microbolometers. TeraFET detectors usually outperform microbolometers at frequencies below 2 THz.

## 2. Detector Design

**Figure 1:**
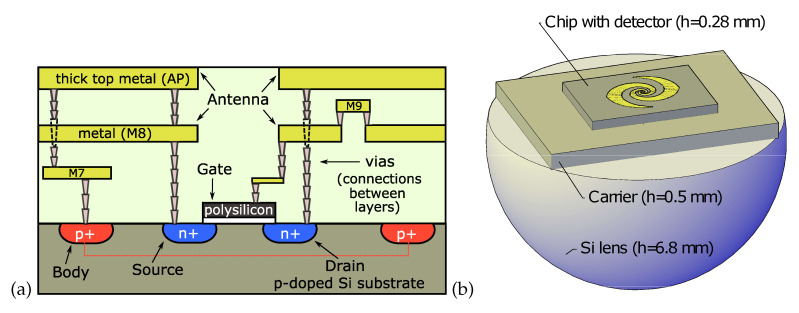
(**a**) Schematic cross-sectional view of the detector chip (not to scale); (**b**) Device with the monolithically integrated log-spiral antenna mounted on a carrier substrate and placed on a hyper-hemispherical lens (diameter of the spherical part of the lens: 12 mm).

The experiments were conducted using a broadband TeraFET detector with a 670-μm-diameter log-spiral antenna. The device was already introduced earlier in Reference [34]. It has an inner radius of 17 μm, an outer radius of 27 μm, and exhibits 1.5 windings. The detector chip was fabricated by a 90-nm CMOS foundry technology. The cross-sectional structure of the detector is shown in Figure 1a. The active region consists of an NMOS FET implemented on a 0.28-μm-thick *p*-doped Si substrate. The transistor is surrounded by a $p^{+}$-type body.

The antenna is built in the back-end of the structure using the M8 metal layer and the thick top metal (AP metal, AP standing for Advanced Packaging). One antenna leaf, fabricated in both the M8 and AP layers, connects through vias with the source contact of the FET. The second leaf is also made from the two metal layers, which are, however, not wired together by vias. The antenna pattern in the AP layer connects with the drain contact of the FET, that in M8 with the gate contact. The small step to M9 (shown in Figure 1a) was required for device fabrication in the foundry in order to limit electrostatic charging of the antenna leaf, which could lead to destruction of the FET by electrostatic discharge through the gate dielectric. This approach for the antenna design provides an elegant way to allow independent DC biasing of the gate and read-out of the rectified signal through the source-drain contacts, and at the same time to achieve a capacitive shunting of drain and gate at GHz and THz frequencies. The AC short is provided by the capacitance of the two drain/gate metal layers of the antenna leaf, and puts the gate and the drain on the same AC potential. This leads to an injection of the THz signal into the FET channel from the source side only, therefore the asymmetry condition required for efficient rectification [35,36] is fulfilled. Both the $p^{+}$ body and the source are connected to external DC ground via the AP layer.

The right side of Figure 1 displays the THz-optical detector assembly (from bottom). The detector is illuminated with the THz radiation impinging through its substrate. The detector chip is glued onto a 0.5-μm-thick piece of a Si wafer serving as a carrier for the chip placement in a PCB holder (the latter is not shown). The holder with the chip is fixed in a detector module which is analogous to the one presented in Reference [37]. It contains a hyper-hemispherical Si substrate lens which is mechanically pressed to the Si carrier with a lens holder. A thin layer of silicone paste improves the refractive index matching, assuring that no air gap is in-between the lens and the Si carrier.

While the detector’s performance in an active illumination scenario was already presented and put into perspective in Reference [34], in the following section we provide additional simulation and measurement data which is of relevance for passive detection. After that we discuss the detector’s properties and extrinsic measures taken for its use in practical system. These measures consist of (a) the direct integration of a low-noise amplifier with low 1/f noise corner frequency, (b) the implementation of electromagnetic shielding of the detector module, and (c) the optimization of the optical coupling using paraboloidal mirrors for the thermal radiation onto the detector.

We add here the information that the speed of response of the TeraFET detector is much higher than that of thermal detectors. The intrinsic speed is determined by the transit-time-limited cut-off frequency of the transistor, which is in the range of tens of GHz. In the present application, the integration time of the lock-in amplifier) determines the speed of response (for values, see below).

## 3. Intrinsic and Optical Detector Parameters of the Broadband TeraFET

The detector was modeled using an analytic hydrodynamic model [38] which describes the rectification in the FET channel in a distributed transmission line picture able to capture the development of charge density waves which propagate along the transistor channel. More details about the modeling of the entire TeraFET (including the radiation coupling to the antenna) and the simulation procedure are described in our previous publications References [34,37,39,40,41,42]. The intrinsic TeraFET parameters were obtained from the measured DC resistance $R_{\mathrm{DC}}$. The extracted parameters are as follows: carrier mobility $\mu =355\;{\mathrm{cm}}^{2}/\mathrm{Vs}$, threshold voltage $V_{\mathrm{th}}=0.42$ V, momentum scattering time τ=52.6 fs, and source contact resistance $R_{s}=69\;\Omega$.

With the device model, we calculated both the intrinsic (electrical) and the optical responsivity ${ℜ}_{I}$ and NEP at a gate voltage $V_{g}=0.45$ V. The determination of the intrinsic quantities assumes all power to be delivered directly to the FET, while the optical quantities are calculated with the inclusion of the optical losses, the radiation coupling efficiency and the losses during signal guiding to the FET [34,37]. The optical losses arise mainly by beam reflection at the Si substrate lens (transmittance: ≈0.7) and by scattering losses (≈0.5). The radiation coupling efficiency is the product of the antenna efficiency (of 0.4) and the Gaussian beam coupling efficiency (of 0.7). The total factor of conversion of optical to electrical power in the antenna is 0.1. For the calculation of the signal transfer to the FET, the mean value of the real part of antenna impedance equal to 85Ω.

The optical NEP of the detector can be experimentally determined from its voltage noise amplitude $V_{N}$, the optical responsivity, and the equivalent noise bandwidth (ENBW) defined by the integration time and the output filter in Reference [43]:

(1) $$\mathrm{NEP}=\frac{V_{N}}{{ℜ}_{V}\sqrt{ENBW}}.$$

Here, ${ℜ}_{V}$ is the voltage responsivity, ${ℜ}_{V}={ℜ}_{I}\cdot R_{\mathrm{DC}}$. Without additional amplification, the output noise of TeraFETs with unbiased channel is fully determined by thermal fluctuations of the detector’s DC resistance $R_{\mathrm{DC}}$ [44].

**Figure 2:**
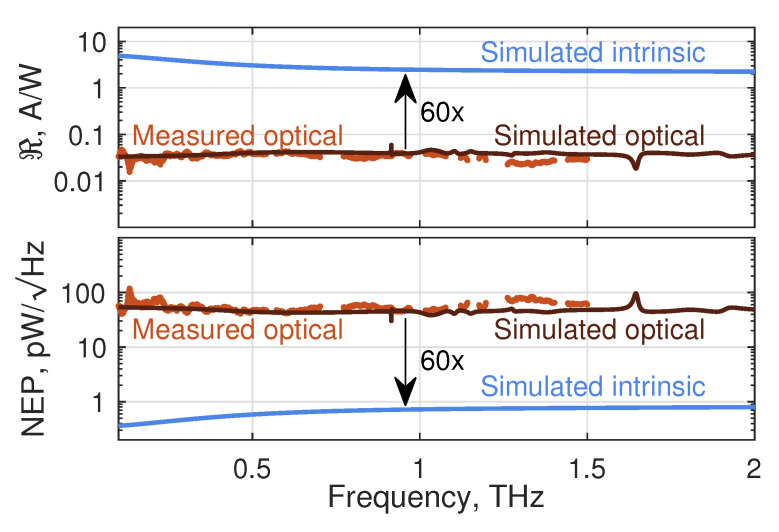
Simulated intrinsic (blue) and optical (black) responsivity and noise-equivalent power (NEP) of the TeraFET with an integrated log-spiral antenna. The brown dots represent the measured data. Black arrows indicate the ratio between the intrinsic and the optical quantities. The calculations cover the range 0.1–2 THz as the simulations for higher frequencies would have required much more computational resources. The experiments were limited upwards to 1.5 THz by the sensitivity range of the pyroelectric and Golay cell power meters used for power calibration.

Results of the simulations (up to 2.0 THz) are shown in Figure 2 and compared with results of the measurements (up to 1.5 THz). The calculated optical ${ℜ}_{I}$ and NEP both exhibit a smooth frequency response, the flatness being more pronounced than that of detectors with a bow-tie antenna reported about in Reference [34]. The experimentally determined frequency dependencies corroborate this remarkable prediction. The agreement is near-quantitative. The model predicts a nearly constant value of the optical ${ℜ}_{I}$ (NEP) of around 40 mA/W (42 pW/$\sqrt{\mathrm{Hz}}$) over the whole frequency range, in good agreement with the experimental data. The measured ${ℜ}_{I}$ values above 1 THz might be slightly underestimated (the NEP overestimated) due to a probable difference of water vapor concentration in the air [15] between the response measurements and the calibration measurements. It should be noted also that we left out data points at the frequency positions of the dominant water absorption lines.

The simulated intrinsic ${ℜ}_{I}$ (NEP) is larger (smaller) than the respective optical quantities by a factor of 60. The large difference underscores the room for improvement of the power transfer from the THz beam to the electrical signal reaching the transistor channel.

## 4. Thermal Sensitivity of the Detector

One of the most important parameters of a detector of thermal radiation is the noise equivalent temperature difference: $\mathrm{NETD}=\frac{V_{N}}{\partial V_{\mathrm{Out}}/\partial T}$. The quantity specifies the smallest temperature difference ∂T sensed by the device, whose field-of-view is filled with black-body radiation inducing the signal change $\partial V_{\mathrm{Out}}$.

**Figure 3:**
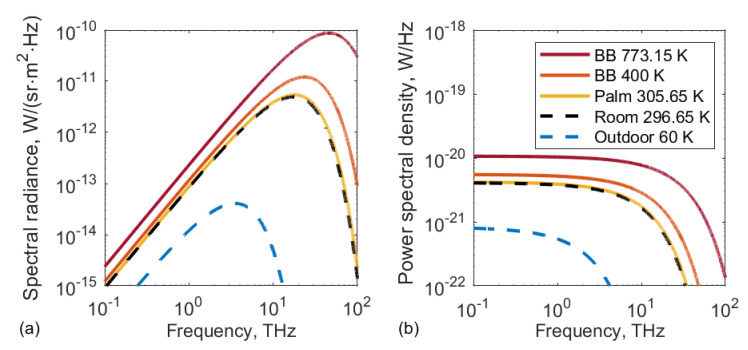
(**a**) spectral radiance of a black-body (BB) emitter at various temperatures; (**b**) and power spectral density received by the ideal antenna (right). Dashed lines show the usual background radiation, indoor (296.65 K, black) and outdoor (sky temperature of 60 K, blue). The solid lines display the BB radiation at the temperatures of the human palm (305.65 K, yellow), of the thermal source used in Reference [33] (773.15 K, red) and of the sensitivity limit at which thermal radiation was still detected in Reference [32] (400 K, orange).

The incoming radiative power from a grey-body source of frequency-dependent emissivity ϵ(f), falling within a solid angle-of-view $\Omega_{A}$ onto a detector with the effective antenna area $A_{e}$, can be directly calculated from Planck’s law for the spectral radiance of black-body radiation (which is depicted in Figure 3a for various temperatures of the radiator):

(2) $$P=\frac{1}{2}\int_{f_{1}}^{f_{2}}\frac{\epsilon \left(f\right)2hf^{3}}{c^{2}}\cdot \frac{\Omega_{A}\cdot A_{e}\left(f\right)}{e^{\frac{hf}{k_{B}T}}-1}\;df\;.$$

Here *c*, $k_{B}$ and *h* are the speed of light, the Boltzmann constant and the Planck constant, respectively. The factor 1/2 accounts for the fact that we assume a polarization-selective antenna and non-polarized thermal radiation. The frequency integration range is defined either by the employed filters or by the bandwidth of the detector’s responsivity.

We can simplify Equation (2) with the Rayleigh-Jeans approximation which holds for frequencies $f<k_{B}T/h\approx 6.15$ THz at room temperature. Additionally, we can use the relationship between effective area of an antenna and its directivity [45,46] to obtain $\Omega_{A}\cdot A_{e}\left(f\right)={\left(c/f\right)}^{2}$. The latter relationship is extremely helpful, as it allows us to make quantitative predictions about the power spectral density available to the detector, without having to consider the physical properties of the antenna. This is fundamentally different from the case of thermal detectors such as Golay cells which integrate impinging radiation power over the detector area.

One finds that the power spectral density available for absorption at the antenna has a frequency dependence as depicted on the right side of Figure 3 for various temperatures of the black-body radiator, assuming ε(f)=1∀f. The important result is that the frequency dependence is flat over a range which depends on the temperature of the radiator. At room temperature, the 3-dB roll-off is at 8 THz, well above the bandwidth of our detector. While this corner frequency is already above the validity range of the Rayleigh-Jeans approximation, one can still draw the conclusion, that any improvement of the detector’s sensitivity to thermal radiation will be proportional to the improvement of its bandwidth (provided the detector’s NEP can be maintained).

Performing the integration over *f* in Equation (2) leads to the simple expression $P=k_{B}T\langle \epsilon \rangle \Delta f$, with $\langle \epsilon \rangle =\left(1/\Delta f\right)\cdot \int_{f_{1}}^{f_{2}}\epsilon \left(f\right)\;df$ and $\Delta f=f_{2}-f_{1}$. Without addional filtering, Δf is the detector’s bandwidth. The proportionality P∼Δf again shows that the power available for absorption by the detector depends on its bandwidth.

From the responsivity ${ℜ}_{V}=V_{\mathrm{Out}}/P$, one obtains the signal change with temperature according to $\frac{\partial V_{\mathrm{Out}}}{\partial T}={ℜ}_{V}\frac{\partial P}{\partial T}={ℜ}_{V}k_{B}\langle \epsilon \rangle \Delta f$. With Equation (1), the NETD can then be expressed in terms of the NEP of the detector:

(3) $$\mathrm{NETD}=\frac{\mathrm{NEP}\cdot \sqrt{ENBW}}{\langle \epsilon \rangle k_{B}\Delta f}\;.$$

Evaluating this expression for the presented detector, the smallest detectable temperature difference turns out to 2.2 K, assuming Δf=1.4 THz, unity emissivity, and an integration bandwidth of 1 Hz. As theory predicts at even larger Δf, the theoretical NETD could be lower.

## 5. Black-Body Radiation Coupling

**Figure 4:**
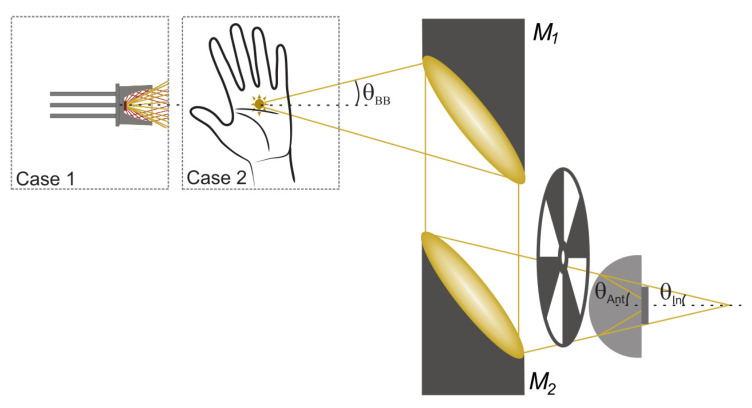
Set-up for the detection of thermal radiation from either the ceramic thermal source or a human palm. The commercially available ceramic radiator comes mounted in the center of a small paraboloidal reflector. $M_{1}$ and $M_{2}$ denote the paraboloidal mirrors. They have equal focal lengths FL; the optical arrangement is in an aberration-free 4-FL optical relay configuration. The thermal signal is either electrically or mechanically chopped at 77 Hz before entering the substrate lens of the TeraFET detector. $\theta_{\;\mathrm{BB}}$, $\theta_{\;\mathrm{In}}=\theta_{\;\mathrm{BB}}$, and $\theta_{\;\mathrm{Ant}}$ denote the beam acceptance angles of $M_{1}$, $M_{2}$ and the antenna.

We performed measurements with both an electrically heated ceramic radiator reaching a temperature of up to 600 °C (however, without specified emissivity for the THz frequency range) and the radiation from a human hand at ambient conditions (room temperature, 33-% air humidity). The grey-body radiation was projected onto the detector unit by two off-axes paraboloidal mirrors. The set-up is depicted in Figure 4. The thermal radiation from a spot of the respective source is collimated by the first mirror and focussed onto the detector by the second one.

**Figure 5:**
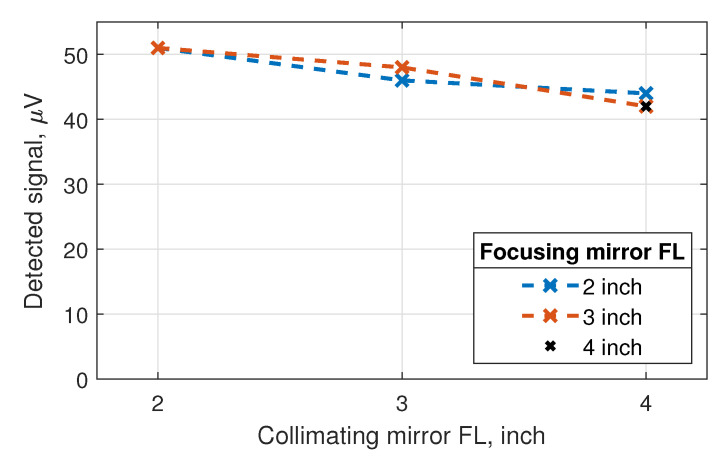
Detected signal for various combinations of paraboloidal mirrors $M_{1}$ and $M_{2}$ with different focal lengths (FLs); for the set-up, see Figure 4. The optimization of the configuration for the best radiation coupling was performed with the electrically chopped ceramic radiator.

For the identification of the best power coupling configuration, we performed a set of experiments with the ceramic radiator, electrically chopped at a frequency of 77 Hz, and tested various combinations of paraboloidal mirrors with reflected focal lengths of 2, 3, and 4 inch (diameter of all mirrors: 2 inch). Results are depicted in Figure 5. The selection of the collimating paraboloid was found to be more critical than that of the focusing mirror. The smaller the focal length of the collimating mirror, the higher the detected signal turned out to be. This can readily be understood, as the collection cone of the paraboloid increases inversely with the focal length. In contrast, the choice of the focal length of the focusing mirror $M_{2}$ was uncritical. Using a 2-inch collimating mirror $M_{1}$, we obtained the same detector signal for the two focal lengths of 2 and 3 inch of $M_{2}$. This means that we covered the radiation profile of the detector’s antenna equally well in both cases.

Another outcome of the study was that the choice of two paraboloids with focal lengths of 3 inch instead of 2 inch comes at a signal penalty of only a few percent. This finding led us to work with these longer focal lengths in the experiments described below. The reason is that all experiments with the human palm as the radiation source and some of those with the ceramic radiator were performed with mechanical chopping, hence needed space for the placement of the chopper, which we mounted between $M_{2}$ and the detector. Note with regard to the experiments with the ceramic radiator that the mechanical chopping was conducted at full thermal source power with a radiator bias voltage of 7 V, while the electrical chopping had to be limited to a 5-V bias modulation (0-V-to-5-V square wave signal) and hence reduced emitted power because of limitations of the signal generator.

In order to prevent ambient noise from entering the detector unit, the detector was enclosed in a 40 × 40 × 80-mm${}^{3}$ metal case comprising a DC-coupled low-noise amplifier with 40-dB gain and 3-MHz bandwidth. The output noise of the detector with the low-noise amplifier was measured to be 1.38 μV/$\sqrt{\mathrm{Hz}}$ above 10 Hz.

The temperature of a human hand in thermal equilibrium with the ambient is specified in the literature to be between 32 °C and 35 °C [47]. During our experiments, we measured the temperature of the palm, which was used as radiation source, with an IR thermometer. The difference between room temperature (23.5 °C) and palm temperature was found to be around 8.7 °C.

## 6. Detection of Radiation from Ceramic Heat Source and Human Palm

**Figure 6:**
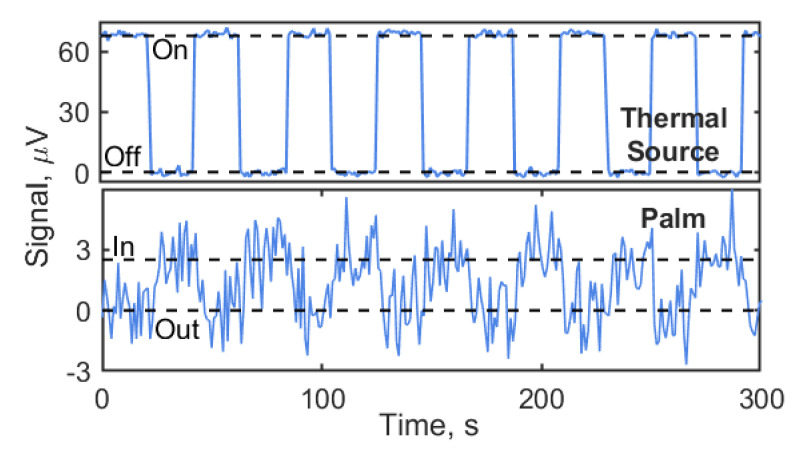
Detection of the radiation from the ceramic thermal source (top) and a human palm (bottom). In both cases, the radiation is mechanically chopped for lock-in detection. On/Off (In/Out) modulation at 25 mHz (period of 40 s). The signal-to-noise ratio (SNR) is 54 for the ceramic source, and 1.97 for the palm. The noise-equivalent temperature difference (NETD) for the latter amounts to 4.4 K.

Figure 6 displays time traces of the detected signal with the radiation repetitively blocked with a period of 40 s (in the case of the palm, the hand was periodically moved away). The measured “On/Off” signal of the ceramic heat source reaching 600 °C temperature (without specified emissivity in THz frequency range) is displayed in the upper panel of Figure 6, the “In/Out” signal of the palm in the lower panel. Both traces were recorded with scan steps of one second, having set the lock-in amplifier to an integration time of 200 ms and an output filter of 12 dB/oct. The corresponding ENBW is 0.833 Hz.

The mean value of the signal from the ceramic heat source in the “On” state equals 69 μV. The root-mean-square (rms) value of the fluctuations is 1.27 μV, which is found to be equal to the rms fluctuations in the “off” signal. The corresponding SNR is 54. The mean value of the “In” signal from the human palm is found to be 2.5 μV, determined as the rms value of the Fourier amplitude at 25 mHz. This corresponds to a SNR of 1.97. With the temperature difference of 8.7 K between palm and the ambient, the NETD is found to be 4.4 K. For a 1-Hz bandwidth instead of the ENBW of 0.833 Hz of the measurements, this translates into a NETD of 4.8 K.

Comparing this value with the expected one of 2.2 K, derived with Equation (3) for 〈ϵ〉=1, one has to first correct for a realistic emissivity. It is well known that the emissivity of human skin is around 0.97–0.99 in the IR frequency range. For GHz and THz frequencies, one finds the following limited information. At GHz frequencies (0.3–300 GHz), the skin’s emissivity rises from 0.4 to 0.8 [48]. This behavior was attributed to the decrease of the dielectric permittivity of biological water. The variation of the skin emissivity among individuals as well as its dependence on gender, provenance, and physical properties such as the body mass index, was measured quantitatively at 90 GHz with a calibrated radiometer on the palms of 60 healthy participants [49] (36 male and 24 female). The mean emissivity of the male palms was found to be 0.451 with a standard deviation of 0.0997. The female palm emissivity was measured to be 0.430, the standard deviation being 0.0951. According this study, the emissivity is higher at higher body mass index and thicker skin. Another study [50] found a higher emissivity of the human palm at around 0.5 THz under mental and physiological activity compared to the resting state. Reference [51] reports three values for the emissivity of the inner arm at 0.1 THz, 0.5 THz and 1.0 THz.

**Figure 7:**
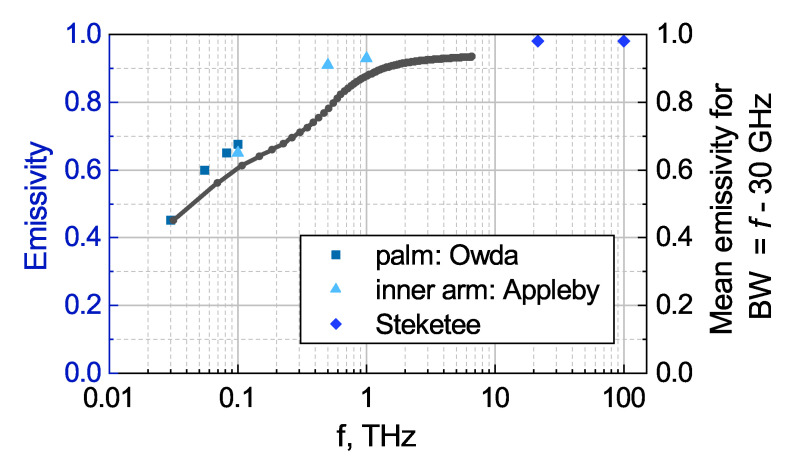
Emissivity of human skin at various frequency ranges, as given in Reference [52] (diamonds), Reference [51] (triangles) and Reference [48] (squares). Grey dots connected by a grey line show the mean emissivity over the bandwidth BW $=f\;-\;f_{0}=f\;-\;30$ GHz, were *f* is the frequency of the *x*-axis, and $f_{0}=30$ GHz is the lower bound of the detection band.

Figure 7 depicts GHz and THz emissivity values of the literature obtained either for the palm or the inner arm, for the IR frequency range, the choice of the region of the body is insignificant. In order to obtain the emissivity values for various detector bandwidths, we linearly interpolated the depicted data points and calculated the mean emissivity value for the bandwidth $\Delta f=f-f_{0}=f$ – 30 GHz, were *f* is the respective frequency value of the *x*-axis and $f_{0}$ is the assumed lower limit of the detection band of the TeraFET as determined by the spectral characteristics of its antenna. A mean emissivity 〈ϵ〉 of 0.9 is obtained for broadband detection with a bandwidth of 1 THz. In the lower frequency bands, however, the radiation will not be emitted so efficiently. For example, in the 30-to-100-GHz window, the mean emissivity is only 0.6. For detection with a larger bandwidth, the calculated 〈ϵ〉 equals 0.81 for f=0.5 THz, 0.9 for f=1.0 THz (as given above), and 0.92 for f=2.0 THz. For the bandwidth of 1.4 THz assumed for our experiment, the corresponding value is 〈ϵ〉=0.915. To give an example of the gain in thermal radiation power available for absorption by the detector, if its bandwidth is increased: Extension of the sensitivity range from the 30-to-100-GHz window to the 30-to-1000-GHz window raises the usable power by a factor of (1000−30)/(100−30)·0.9/0.6≃21. This estimate is based on the flatness of the power spectral density of Figure 3b.

Coming back to the comparison of the measured and the calculated NETD, there is a second correction factor to be taken into account. One has to consider that the measured signal is square-wave-modulated by the mechanical chopping (see Figure 6), which decreases the detected signal by a factor of $\pi /\sqrt{2}$ compared with the unmodulated case. With both corrective terms, the expected NETD is hence 4.65 K for an ENBW of 0.833 Hz, respectively 5.1 K for 1 Hz, in excellent agreement with the experimentally determined values.

## 7. Passive Imaging of the Human Hand

**Figure 8:**
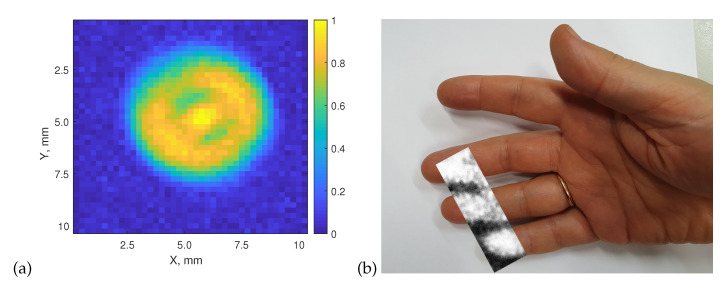
Thermal images recorded with the broadband Si CMOS TeraFET detector at room temperature. (**a**) image of the thermal emission pattern of the heated ceramic source; (**b**) image of sections of three fingers of a human hand. The image is superimposed on a photograph of the hand. In both cases, mechanical chopping of the thermal radiation was applied.

We now come to two examples of thermal imaging by raster scanning, one recorded at elevated object temperature and the other at human-body temperature. The images are displayed in Figure 8. The left side shows a measurement of the emission pattern of the ceramic radiator (fully heated at a bias voltage of 7 V). The data acquisition time was 28 min 40 s, 40×40 pixels were recorded with a pixel pitch of 0.25 mm. The image reveals strong emission from the center of the device, but also shows pronounced radiation arriving from its perimeter. This is radiation deflected into the beam path by the cup-like reflector into which the radiator is embedded, as shown in Figure 4, Case 1.

Finally, we demonstrate passive imaging of parts of three fingers of a hand (where the temperature is likely to be even lower than in the palm, for which we measured a temperature of 32.5 °C). Figure 8 (right) displays the two-dimensional image as an overlay of a VIS photograph of the whole hand. The hand was placed onto an xy-translation stage and moved at a fixed speed of 1 pixel/s at a step width of 1 mm in both directions, covering an area of 2.5 × 7.5 cm${}^{2}$ (image acquisition time of about 30 min). The settings of the chopper and the lock-in amplifier were as in the experiments described above. Imaging speed and resolution were the same as in Reference [33]. One can clearly distinguish the fingers and the cooler regions in-between.

## 8. Discussion

The predicted NETD for this detector is 2.2 K for a 1-Hz effective noise bandwidth and unity emissivity. Using lock-in detection at an effective noise bandwidth of 0.833 Hz and assuming a realistic emissivity of the skin, the expected NETD is 4.65 K, low enough to detect the 8.7 K temperature difference of a human hand against a room-temperature background, and to record images by raster-scanning. The experiments yielded a measured NETD of 4.4 K, in excellent agreement with theory. There is still a lot of room for improvements in the radiation coupling efficiency which should allow to further increase the detector performance.

Over the last years, the sensitivity of TeraFETs has continued to approach the level needed for the detection of the black- and grey-body radiation from warm and moderately hot objects while maintaining the detector itself at room temperature. Until recently, TeraFETs were cooled to cryogenic temperatures for the passive detection of objects [33]. The improvements of the sensitivity and especially of the detection bandwidth has now led to the present proof of concept of passive imaging with uncooled TeraFET detectors.

While it is remarkable that a temperature difference of only 8.7 K can be detected against a room-temperature thermal background, there is clearly the need to improve the detector’s performance further in order to allow for much faster data acquisition. The most direct ways to enhance the performance are the increase of the bandwidth and of the NEP of the detector. Any improvement of either of them by a factor of two reduces the NETD by a factor of two or the time constant required for signal averaging by a factor of four. However, one should not forget, that such improvements are also beneficial for measurements with cooled detectors. For our devices, we find an improvement of the NEP by a factor of 3.8 upon cooling to 77 K [53]. The NEP scales with an inverse proportionality to the temperature from 300 K to 77 K (some authors report even more pronounced improvements for their transistor devices [54,55]. This improvement implies that we could reach an NETD of 1.2 K for an ENBW of 0.833 Hz with the broadband Si CMOS TeraFETs discussed here. With the thermal emitter heated to 773 K, one could then record an image with a dynamic range of 23 dB for the same data acquisition time as that of Figure 6. As the detectors can be easily produced in multi-pixel arrays [56,57], novel camera applications of thermal vision in the lower THz frequency range may become feasible.

## 9. Conclusions

Here we demonstrate passive THz imaging using only the thermal radiation of a human body to illuminate a broadband Si CMOS TeraFET detector at room temperature. To our knowledge, this is the first demonstration of passive imaging with any broadband uncooled detector in the low THz frequency range. The TeraFET detector with an integrated log-spiral antenna exhibits a flat optical responsivity around 40 mA/W over the 0.1–1.5 THz range (optical NEP: 42 pW$/\sqrt{\mathrm{Hz}}$). Simulations suggest an even wider flat-responsivity range extending above 2.0 THz. The measured NETD is 4.4 K (with a noise bandwidth of 0.833 Hz). This is in good agreement with the predicted NETD of 4.65 K.

## Abbreviations

The following abbreviations are used in this manuscript:

TeraFET	field-effect transistor-based THz detector
CMOS	complementary metal-oxide-semiconductor technology
NETD	Noise-equivalent temperature difference
NEP	Noise-equivalent power
SNR	signal-to-noise ratio
ENBW	equivalent noise bandwidth
